# What Should Be the Fee for a Denture?

**Published:** 1916-05

**Authors:** W. J. Holroyd


### WHAT SHOULD BE THE FEE FOR A DENTURE?

BY W. J. HOLROYD, D.D.S.

Financial success is all a matter of educating the patient and studying costs, and raising fees so one can buy these new labor saving appliances and become up to date. Remember that the manufacturer is the man who places the price on everything in your office. You were told the price and you paid or left it. But the majority of dentists allow their patients to quote the price of work. We are not salesmen, just mere order takers. Patients say our fee is too much and we cut the price to suit their pocketbooks—in the face of the fact that they are paying other manufacturers $1 where we get 25 cents.

Now it has been proven that to maintain an office on an efficient basis and constantly keep in touch with new improvements, takes 50 cents on the dollar of gross receipts at present average fees, before the dentist has anything left for himself. To make a larger percentage on the dollar he will have to charge more all along the line for his work. To do this intelligently he will have to go to the trouble of "timing" himself during different operations. If he does that, he will not need any one to beg him to change his viewpoint.

All business men and manufacturers put a price on their product, based on *quality* of *labor* and the *time* it takes, and no trouble is too great that enables them to get results. They sit up nights and have meetings and call in cost experts to help them. This brings us to the analysis of the heading of this paper.

What should we conscientiously charge for plates—what should be the minimum price?

Some time ago we sent broadcast to 35,000 dentists, through the courtesy of *Oral Hygiene*, a time chart for plates, asking dentists to fill in the time required for the different steps and return to us for compilation and averaging. We have received a great deal of information and this article will give you the results. Although it emphatically demonstrates that the dentists need to be taught how to do things for their own good, some men must have thought the timing was intended to be a race—to see how quickly they could do a certain specified piece of work—and some again have been very conservative and have gotten together a very fair average of "times."

The timing of operations means an average of many times (say 20), and when a man writes that he can paint an impression, pour model and mix his plaster right, so that it has no bubbles and every particle of plaster comes in contact with water in two minutes and then separate and build up trial base plate, according to the latest accepted standard, in *three* minutes, and at *this* rate make a whole upper denture in one hour, forty-seven minutes, then his records are not to be put down to average with other conservative men who do the work more thoroughly. Such timing is not correct and it is a reflection on the compiler's intelligence to quote his figures in averaging.

The average time from more than 150 reliable statistics results as follows:

### CHAIR TIME.

Time consumed in making contract for plate:

| | | |
|---|---|---|
| Examination and consultation | 30 | minutes |
| Taking impression | 15 | " |
| Taking bite | 20 | " |
| Trial plate fitted | 30 | " |
| Fitting denture in mouth | 15 | " |
| There is an average of 4 trips for plate to be adjusted, sometimes to be scraped and troubles at other times imaginary, but consuming time about 20 minutes each visit | 80 | " |

### LABORATORY TIME.

| | | |
|---|---|---|
| Painting impressions and pouring model | 12 | minutes |
| Separating and making trial base plate | 20 | " |
| Mounting on articulator | 10 | " |
| Selecting teeth | 15 | " |
| Articulating | 46 | " |
| Final waxing | 25 | " |
| Investing | 20 | " |

| | | |
|---|---|---|
| Packing, putting in and taking from vulcanizer. (Does not include time for actual vulcanizing) ............ | 90 | minutes |
| Scraping and polishing ............................ | 60 | " |
| | 60)488 | " |
| | 8 hrs. | 8 min. |
| Percentage of makeovers 25 per cent................. | 2 | " |
| | 10 | " |

These averages are compiled from quite a number of dentists and prove that work can be averaged to such an extent that a fee basis can be arrived at. Remember, this sending out to 35,000 dentists for data was never done before, and proved quite a task, and if any skeptics differ with any of the above statistics, I shall be glad to be corrected if they will go to the same amount of trouble.

Now if it takes 50 cents on the dollar to conduct an up-to-date office and if you only charge $20 for a plate and it takes you ten hours to make it, you can readily see that it costs you $10 to make that plate, counting material and overhead expenses (which lots of dentists never think about), leaving practically $1 per hour for yourself, which sum a jobbing plumber demands when he comes to open your sink. A tile setter gets 62½ cents per hour. After spending your money and three years for education and taking all the responsibilities of business worries and building up your practice, ask yourself if you are not worth more than $1 per hour net. Some of the mechanics' wages are only a few cents less per hour and they have no overhead responsibility.

You might say the laboratory does a lot of it and it is really not your time. I can get a plate made for $3 and charge $15, that is $12 profit. This way of figuring is not right. Too many dentists figure that way. Overhead charges and running expenses must be put against the first price, and considering that the dentist is made responsible for any work that does not fit and any makeovers must come out of his own pocket, he must charge and receive the profit as if he had made the whole plate himself. *If the*

*dentist has to stand the brunt of mistakes of other people whom he employs, he at least is entitled to the profits of their labor.* It is so all over the commercial world. A dentist of ten years' practice should be earning $6 per hour gross—earning $3 net per hour.

Another point of vital importance. It has been conceded, after a lengthy canvass of the foremost men of the profession and accountants and cost experts on the side, that highly specialized laboratory work should command as much pay as work at the chair.

This special problem of whether the laboratory charges should be charged at the same price as chair time was put up to the representative of one of the largest cost expert firms of Pittsburgh, who handles the business of large corporations, and his decision, after giving the point much thought, was decidedly in the affirmative. Large law firms do it, the work of subordinates is charged the head of the firm's prices, and the head of the firm takes the responsibility. The principle is the same in our case. Other instances can be given in architects' and engineers' offices, etc., which proves enough precedent, and considering that these cost experts have studied such points as these and that these decisions are accepted by banks, and the commercial world in general, we are only showing our ignorance by doing otherwise. Such a course will only prove our undoing.

It has also been brought out in another paper that the productive time in an office for one year is only *1,000* hours; the dentist may be in his office 2,500 hours but the losses, including time exceeding that contracted for, makeovers, times for consultations and charity work—cut down the actual producing hours to an alarming extent. One thousand hours have to stand the brunt of the charges.

Therefore, the foregoing brings us to the crucial point that ten hours' work on a single vulcanite plate at $6 per hour is $60, which is the minimum price that ought to be charged, gold plates in proportion, considering the knowledge which is put into a properly fitting denture. This leaves $30 for yourself.

This price, gentlemen, is what single rubber dentures will bring when the dentists wake up to the cost of conducting their practices and a knowledge of what is their due. There are more dentists who have realized these facts and are charging these prices than you would imagine, and at that, the patient is not paying one whit more than he pays for other things, considering value dollar for dollar. The poor man who scrapes his money together and pays $250 for a piano pays the salesman $125 profit. If it takes the salesman one hour to sell him that piano that workingman pays him $125 for one hour's work and that salesman does not shed any tears over it either. The poorest working people can and do afford and pay the various dealers a bigger rate of profit for various articles than they do the dentist, considering the length of service rendered. It is estimated that the dentist only asks 25 cents on the dollar compared with other men in other business. Yes, and even less. Instead of being called D.D.S. we ought to be called D.P.P.—Department of Public Philanthropy.

It is not just, considering the knowledge required, that a dentist should receive only the pay of a good workingman or artisan. This fact explains, as a Dean of a college once said, why after five years a good many graduated dentists drop out of practice into other callings. A good many dentists in the farming districts, who complain of cheap prices, claim their class of patients could not pay these prices, *but do you find the merchants of the city reducing prices of farmers' necessities on that account?* The farmers pay the merchant the same price and ratio of profit as does the workingman in the city. Every other line of business from pins to automobiles is now controlled by interests that have educated (a little at a time) the masses (including dentists) to pay considerably more than was paid ten years ago. That is an accepted fact, but the fees for dental services have not changed to an appreciable extent in the last fifteen years.

It bespeaks a lack of business acumen and a laxity in keeping up with the times on the dentists' part. Dentists may protest and say proper fees can't be gotten, but they are being gotten by the ones that are "waking up," who are determined to secure a competence in their old age.

A word about this competence. A competence is at least $30,000, which yields, at 4 per cent., $1,200 yearly, or $100 a month. Ask yourself, have you got it or are you saving it? If you are saving anything at all it is not from making plates, and it behooves you to study which department of practice you are losing money in, and plates is one of them.

The remedy is—start to-day. When the next patient for a plate comes to you, do not give him any limit, say (to start the propaganda) that he can have any price up to $60, and then he will naturally want to know the difference, then show him the new teeth and work the salesmanship stunt just as it is worked on you. Don't keep on quoting $10, $12, and $15 eternally. Sell service and you will be surprised within six months what a material change you can bring about among the very people you are now working for. Start today and then shortly you will be able to fit up an office such as you have dreamed of—and keep things going along these lines.—*Dental Digest.*

---

I wisht I was a little rock
A-sittin' on a hill;
A-doin' nothin' all day long
But just a-sittin' still.
I wouldn't eat, I wouldn't drink,
I wouldn't even wash,
I'd set and set a thousand years
And rest myself, by gosh.

—*Boston Transcript.*

